# Analysis of diet-induced differential methylation, expression, and interactions of lncRNA and protein-coding genes in mouse liver

**DOI:** 10.1038/s41598-018-29993-4

**Published:** 2018-08-01

**Authors:** Jose P. Silva, Derek van Booven

**Affiliations:** 1 Department of Psychiatry and Behavioral Sciences, Miller School of Medicine, University of Miami, Miami, FL 33136 USA; 2 John P Hussman Institute for Human Genomics, Miller School of Medicine, University of Miami, Miami, FL 33136 USA

## Abstract

Long non-coding RNAs (lncRNAs) regulate expression of protein-coding genes *in cis* through chromatin modifications including DNA methylation. Here we interrogated whether lncRNA genes may regulate transcription and methylation of their flanking or overlapping protein-coding genes in livers of mice exposed to a 12-week cholesterol-rich Western-style high fat diet (HFD) relative to a standard diet (STD). Deconvolution analysis of cell type-specific marker gene expression suggested similar hepatic cell type composition in HFD and STD livers. RNA-seq and validation by nCounter technology revealed differential expression of 14 lncRNA genes and 395 protein-coding genes enriched for functions in steroid/cholesterol synthesis, fatty acid metabolism, lipid localization, and circadian rhythm. While lncRNA and protein-coding genes were co-expressed in 53 lncRNA/protein-coding gene pairs, both were differentially expressed only in 4 lncRNA/protein-coding gene pairs, none of which included protein-coding genes in overrepresented pathways. Furthermore, 5-methylcytosine DNA immunoprecipitation sequencing and targeted bisulfite sequencing revealed no differential DNA methylation of genes in overrepresented pathways. These results suggest lncRNA/protein-coding gene interactions *in cis* play a minor role mediating hepatic expression of lipid metabolism/localization and circadian clock genes in response to chronic HFD feeding.

## Introduction

More than 70% of the mammalian genome is transcribed as non-coding RNA (ncRNA) while only 1–2% of the mammalian genome is transcribed as protein-coding RNA^1–3^. NcRNAs can be classified as short and long ncRNAs (lncRNAs), which are defined as being shorter or longer than 200 bases, respectively^4^. According to current GENCODE nomenclature, lncRNAs can be further subclassified as: (i) antisense RNAs, which are transcribed from the opposite DNA strand of a protein-coding gene with intronic and/or exonic overlap; (ii) long intergenic non-coding RNAs (lincRNA); (iii) sense-intronic RNAs originating from an intron of a coding gene on the same strand with no exonic overlap; (iv) sense-overlapping RNAs containing in its introns a coding gene on the same strand with no exonic overlap; and (v) 3′-overlapping ncRNAs, which are transcribed from the 3′ untranslated region (3′ UTR) of a coding gene on the same strand. LncRNAs can be spliced, polyadenylated and capped^4^. They are predominantly located in the cell nucleus and usually expressed at much lower levels than protein-coding RNAs. LncRNAs also tend to display a low degree of sequence conservation across species, although lincRNAs with strong sequence conservation have been reported^5,6^. Expression of lncRNAs is cell type-specific and limited to specific developmental time windows^5,7^. LncRNAs can increase or decrease expression of protein-coding transcripts *in cis*^3,5,7^ and *in trans*^8^, through epigenetic, transcriptional and post-transcriptional mechanisms. LncRNAs can interact with histone-modifying protein complexes such as the Trithorax group complex and the Polycomb repressive complex 2^9^ or with DNA methyltransferases^10,11^ to activate or silence genes. Moreover, they can alter splicing, editing or stability of protein-coding RNAs^12^. The importance of lncRNAs in gene regulation is showcased in key biological processes such as X-chromosome inactivation in females, imprinting of parental genes, regulation of stem cell pluripotency, cell differentiation and proliferation, silencing of tumor suppressor genes, DNA damage and cellular stress responses^9,12^.

RNA-sequencing (RNA-seq) is a sensitive and versatile genome technology for profiling lncRNA expression. RNA-seq is superior in terms of detection, resolution and quantification of very low abundance transcripts compared to oligonucleotide-based microarrays^13^. Unlike conventional oligonucleotide-based microarrays, RNA-seq does not use a pre-defined set of complementary oligonucleotide probes for transcript identification. Instead the sequencing reads are reassembled to produce high resolution transcripts under consideration of the most recent sequence annotations. Furthermore, RNA-seq allows simultaneous quantification of protein-coding and non-coding antisense transcripts originating from complementary DNA strands.

Characterisation of functional diet-induced interactions between lncRNAs and coding genes is of interest for identifying novel genomic biomarkers and therapeutic targets for metabolic diseases. Here, we applied RNA-seq to explore the possible existence of *cis*-regulatory interactions of lncRNAs with protein-coding genes in mouse livers in response to chronic HFD feeding. Moreover, 5-methylcytosine DNA immunoprecipitation sequencing (5meDIP-seq) and targeted bisulfite sequencing (BS-seq) determined DNA methylation of protein-coding genes as a possible epigenetic endpoint of *cis*-acting lncRNA/coding gene interactions.

## Results

### Composition of the mouse liver transcriptome

**Figure 1:**
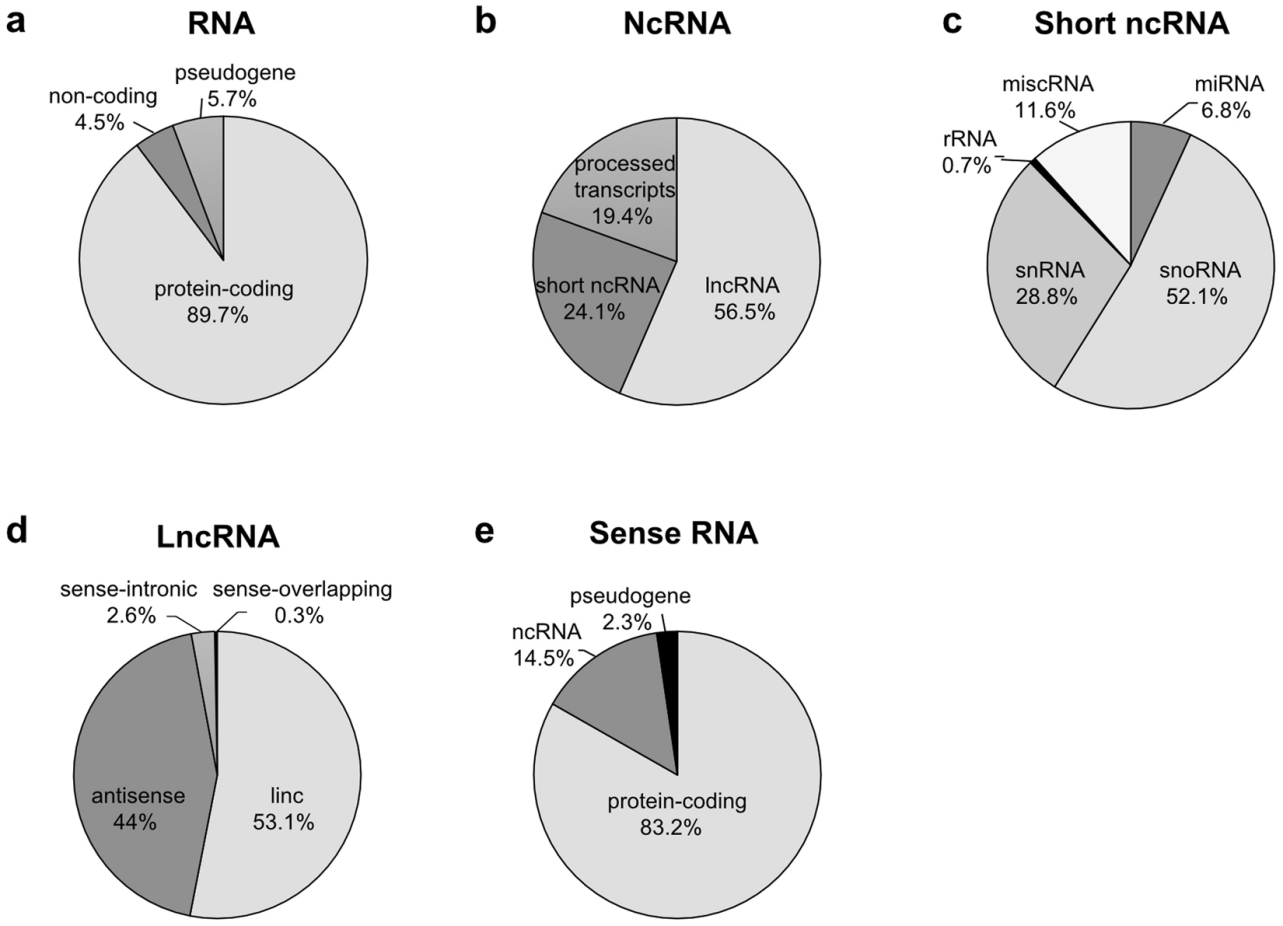
Composition of the mouse hepatic transcriptome by total RNA (**a**) ncRNA (**b**) short ncRNA (**c**) lncRNA (**d**) and sense RNA (complementary to antisense RNA) (**e**). Only RNAs with average expression levels >1 CPM were considered for analysis.

8-week old C57BL6/J male mice were subjected to a STD (n = 6) or cholesterol-rich Western-style HFD (n = 6). After 12 weeks on diet, the animals were euthanised and their livers collected. Directional RNA-seq libraries were generated from 3 pools of 2 mouse livers per diet and paired-end sequenced. RNA-seq produced on average 67 million pass-filter read-pairs per library (HFD: 68.96 million; STD: 64.4 million) (Table 1). Read-pairs were aligned to the mouse genome by the STAR aligner^14^. On average, 51.1 million (HFD: 53.1 million; STD: 49.1 million) or 76.6% (HFD: 76.94%; STD: 76.31%) pass-filter read-pairs mapped to a unique site of the mouse genome with less than 2 mismatches and were used for further analysis (Table 1). The aligned read-pairs were run through HTSeq against the mouse mm10 reference genome for determination of read-pair counts by genes^15^. A total of 13365 genes with average expression levels across all RNA pools >1 CPM were considered for further analyses (Table S1). These genes consisted of 11993 protein-coding genes, 765 pseudogenes, and 607 ncRNA genes (Fig. 1a). NcRNA genes comprised 146 short ncRNA genes, 343 lncRNA genes, and 118 processed transcripts (Fig. 1b). The number of short ncRNAs is likely underestimated because generation of sequencing libraries excluded RNA molecules shorter than 100 nucleotides. Short ncRNA genes comprised 76 small nucleolar RNA (snoRNA) genes, 42 small nuclear RNA (snRNA) genes, 10 microRNA (miRNA) genes, 1 ribosomal RNA (rRNA) gene, and 17 miscellaneous RNA (miscRNA) genes (Fig. 1c). LncRNA genes comprised 182 lincRNA genes, 151 antisense RNA genes, 9 sense-intronic RNA genes, and 1 sense-overlapping RNA gene (Fig. 1d). Antisense RNA genes overlapped 144 protein-coding genes, 25 ncRNA genes and 4 pseudogenes (Fig. 1e).

**Table 1:** RNA-seq alignment statistics.

Sample	Input read pairs	Uniquely mapped read pairs	Uniquely mapped read pairs (%)
HFD A	64811482	47999627	74.06%
HFD B	75925925	58699825	77.31%
HFD C	66170800	52564490	79.44%
STD A	62155721	47486909	76.40%
STD B	75900043	57932416	76.33%
STD C	55098436	41984291	76.20%
Average	66677068	51111260	76.62%
Average HFD (n = 3 pools)	68969402	53087981	76.94%
Average STD (n = 3 pools)	64384733	49134539	76.31%

Three pools of 2 livers per diet (HFD A-C; STD A-C) were paired-end sequenced on the Illumina platform. Input read pairs passed Illumina’s internal quality control filters. Uniquely mapped read pairs are read pairs aligning to a unique site of the mouse genome with less than 2 mismatches.

### Differential gene expression analysis

**Figure 2:**
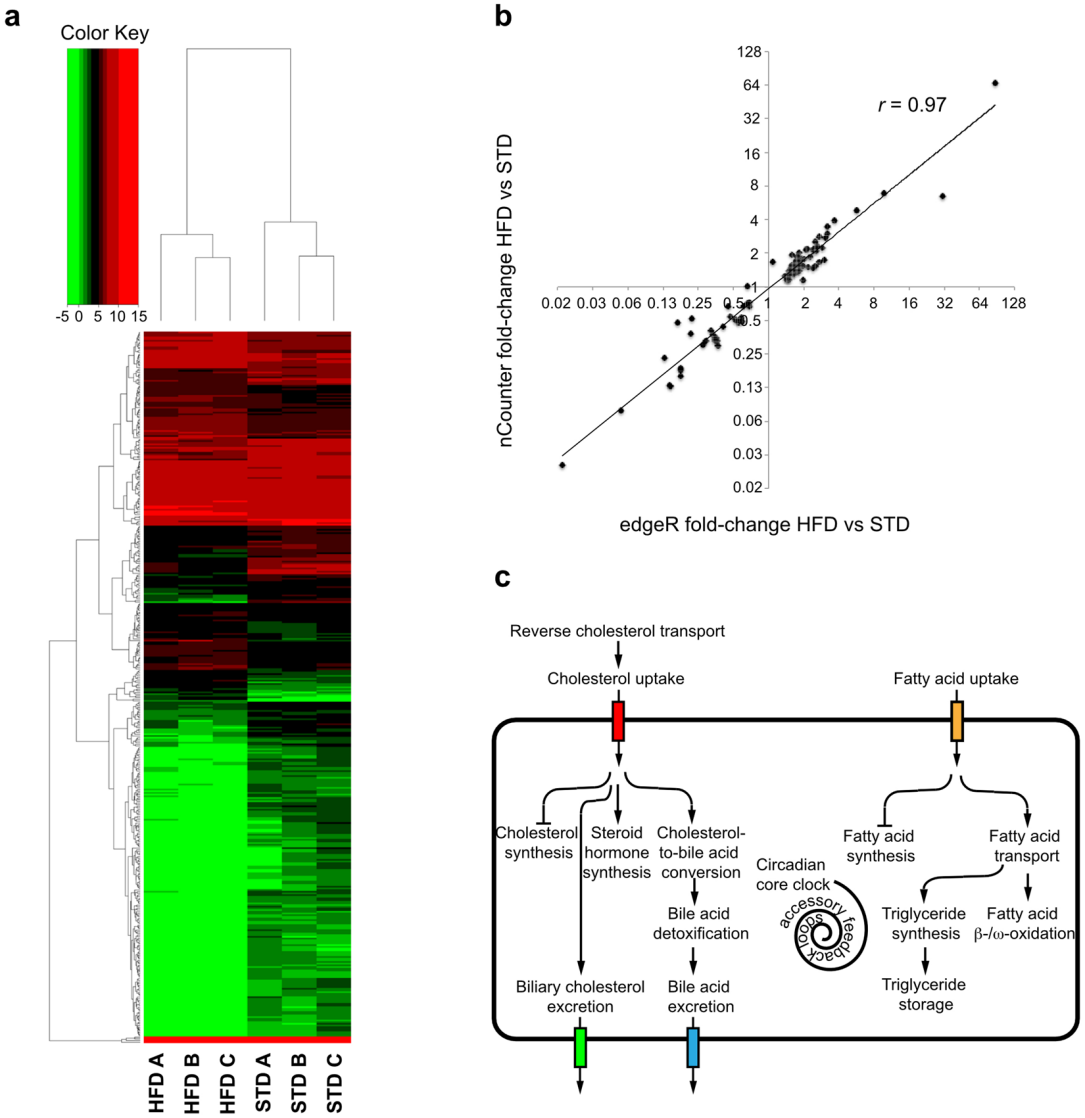
Differential gene expression analyses by edgeR and validation of expression differences by nCounter® technology. (**a**) Two-way clustering of differentially expressed genes by edgeR separates HFD- from STD-fed mouse liver pools. The color code reflects the expression value of a gene given as the log_2_ of counts per million (log_2_CPM). Only genes with average expression levels across all samples >1 CPM were considered for analysis. (**b**) Correlation analysis of fold-changes determined by edgeR and nCounter® technology for 95 protein-coding genes reveals tight concordance (Pearson’s correlation coefficient *r* = 0.97). (**c**) Biological processes modulated by HFD-induced protein-coding gene expression.

The edgeR bioconductor package^16^ determined differential gene expression. Only genes with average expression levels across all samples >1 CPM were considered for analysis. EdgeR yielded 425 differentially expressed genes (DEG) with False Discovery Rate (FDR)-adjusted *p*-values < 0.05 (Table S2). DEG comprised 395 protein-coding genes, 6 lincRNA genes, 7 antisense RNA genes, 2 processed transcript genes, 13 pseudogenes, and 2 snRNA genes. Hierarchical clustering clearly separated the gene expression signatures of HFD and STD livers (Fig. 2a). nCounter® (Nanostring) technology, a digital barcoding detection system for multiplexed expression analysis of RNAs^17^, validated expression of 95 protein-coding genes in individual mouse livers (n = 6 per diet). nCounter® technology confirmed edgeR-calculated fold-change values for most examined protein-coding genes (Pearsson’s correlation coefficient *r* = 0.97; Fig. 2b and Table S3).

### Virtual deconvolution of hepatic cell type composition

To exclude that gene expression changes were related to differences in hepatic cell type composition, we estimated cell type frequencies by deconvolving cell-type specific marker gene expression (Table 2) using the Digital Sorting Algorithm (DSA)^18^ and the meanProfile algorithm in the CellMix1.6 package^19^. The estimation of cell type frequencies focused on hepatocytes, Kupffer cells, and sinusoidal endothelial cells, which represent 52%, 18%, and 22%, respectively, of the number of cells in the mouse liver^20^. Other resident cell populations such as hepatic stellate cells and cholangiocytes were not considered for analysis since their contribution to the total hepatic cell number is below 10% at which point they cannot be estimated accurately^18^. Importantly, inflammatory cell infiltrates and fibrosis as factors influencing cell type frequencies in HFD livers were excluded by histological analysis, which showed a centrolobular micro- and macrovesicular lipidosis (Fig. S1). Both DSA and meanProfile algorithms estimated similar proportions of hepatocytes, Kupffer cells and sinusoidal endothelial cells in both STD and HFD livers (Table 2). These results suggested similar cell type composition in HFD and STD livers.

**Table 2:** Estimation of hepatic cell type proportions by deconvolution analysis.

Algorithm	Cell type	STD Mean (%)	STD SEM (%)	HFD Mean (%)	HFD SEM (%)	*p*-value *t* test
DSA	Hepatocytes	49.7	0.3	50.3	0.3	0.23
DSA	Kupffer cells	25.2	0.2	24.8	0.2	0.23
DSA	Sinusoidal endothelial cells	25.2	0.2	24.8	0.2	0.23
meanProfile	Hepatocytes	44.4	0.3	44.2	0.3	0.51
meanProfile	Kupffer cells	28.3	0.1	28.4	0.2	0.44
meanProfile	Sinusoidal endothelial cells	27.2	0.1	27.2	0.0	0.88

The Digital Sorting Algorithm (DSA) and meanProfile algorithm in the CellMix1.6 package estimated frequencies of hepatocyte, Kupffer cells and sinusoidal endothelial cells by deconvolving cell type specific marker gene expression. The p-values are from two-sided t tests comparing the estimated frequencies of a given cell type in STD livers versus HFD livers.

### Pathway analysis of differentially expressed genes

We conducted pathway analysis of the 425 DEG using DAVID (Database for Annotation, Visualization and Integrated Discovery)^21^ and the BiNGO add-on application to Cytoscape 3.2^22^. DAVID and BiNGO/Cytoscape3.2 mapped 390 and 350 DEG, respectively, to biological processes. Both uncovered a significant overrepresentation of DEG in steroid biosynthesis, cholesterol metabolism, fatty acid (FA) metabolism, lipid localization, and circadian rhythm (Table 3).

**Table 3:** Pathway analysis.

Biological Process	GO-ID	FDR DAVID	FDR BiNGO/Cytoscape3.2	DEG DAVID	DEG BiNGO/Cytoscape3.2
Steroid biosynthetic process	6694	1.6E-13	6.2E-21	20	21
Cholesterol metabolic process	8203	1.0E-10	1.42E-22	17	22
Fatty acid metabolic process	6631	6.8E-04	3.1E-08	16	17
Lipid localization	10876	5.6E-03	1.3E-05	12	11
Circadian rhythm	7623	6.1E-02	8.01E-04	6	6

DAVID and BiNGO/Cytoscape3.2 determined statistical overrepresentation of biological processes using the Benjamini-Hochberg method. Only DEG with average expression levels across all samples >1 CPM were considered for enrichment analysis. Biological processes are denoted by their official gene ontology names. GO-ID refers to the official Gene Ontology Identity Number. FDR DAVID and FDR BiNGO/Cytoscape3.2 refer to the False Discovery Rate-adjusted p-value calculated by DAVID and BiNGO/Cytoscape3.2, respectively. DEG DAVID and DEG BiNGO/Cytoscape3.2 refer to the number of DEG assigned by DAVID and BiNGO/Cytoscape3.2, respectively, to a biological process.

#### Steroid biosynthesis and cholesterol metabolism

DAVID and BiNGO/Cytoscape3.2 mapped 20 and 21 DEG functioning in steroid biosynthesis (GO-ID: 6694), as well as 17 and 22 DEG functioning in cholesterol metabolism (GO-ID: 8203) (Table S4). In addition, we identified 6 unmapped DEG functioning in cholesterol metabolism (Table S4). HFD-feeding lowered expression of numerous genes of the cholesterol synthesis pathway. Consistent with previous gene expression studies of livers of HFD-fed C57BL/6 mice, the transcript levels of the cholesterol synthesis genes *Cyp51*^23–25^, *Dhcr7*^23–25^, *Fdft1*^23,26^, *Fdps*^23–25,27^, *Hmgcr*^23,24^, *Hmgcs1*^23,24,26^, *Hsd17b7*^23^, *Idi1*^24–26^, *Lss*^23^, *Mvd*^23^, *Mvk*^23^, *Msmo1*^23–26^, *Nsdhl*^23–27^, *Pmvk*^23,24^, *Sc5d*^23^, *Sqle*^23,24,26,27^, and *Tm7sf2*^23–25,27^ were significantly decreased in HFD livers. Since Srebf1 and Srebf2 activate transcription of cholesterol synthesis genes and this activation is blocked by excess cholesterol, we scanned these differentially expressed cholesterol synthesis genes for putative Srebf1 and Srebf2 transcription factor binding sites using the Search Motif Tool in the Eukaryotic Promoter Database (EPD)^28^. Most of these genes contained predicted Srebf1 and Srebf2 binding sites in their promoters, −5000 bp to +1000 bp relative to the transcription start site and many of these binding sites were shared between Srebf1 and Srebf2 (Table S12). To support these predictions experimentally, we searched for peaks from mouse Srebf1 chromatin immunoprecipitation sequencing (ChIP-seq) experiments in the Gene Transcription Regulation Database (GTRD)^29^. We found Srebf1 peaks overlapping the promoters of most differentially expressed cholesterol synthesis genes in liver and other tissue samples (Table S12), supporting the notion that these genes have functional Srebf1 and Srebf2 binding sites. Further in line with previous studies, HFD-feeding also led to gene expression changes promoting hepatocellular cholesterol uptake, namely upregulation of *CD36*^23,25,30^ and down-regulation of *Pcsk9*^24^. *CD36* encodes a high affinity receptor for the cellular uptake of cholesterylester from high density lipoprotein (HDL). *Pcsk9* encodes a protein that mediates degradation of the low-density lipoprotein receptor (LDLR). Its downregulation in HFD livers is expected to increase hepatic LDLR levels and consequently hepatocellular cholesterol uptake as earlier shown in *Pcsk9* knockout mice^31^. Further in line with previous studies, HFD feeding also up-regulated transcript levels of the following genes mediating hepatocellular cholesterol efflux, cholesterol to bile acid conversion, bile acid detoxification and bile acid excretion: (i) *Abca1*^23,25,30^ encoding an ATP-binding cassette (ABC) transporter for the hepatocellular efflux of cholesterol onto apolipoprotein A 1 (Apoa1) for HDL formation; (ii) *Cyp7a1*^24^ catalyzing a rate-limiting step in the conversion of cholesterol to bile acid; (iii) the nuclear receptors *Nr1i2* (*Pxr*) and *Nr1i3* (*Car*)^32^ driving transcription of bile acid detoxification and excretion genes; iv) *Cyp3a11*^24,26,27,32^ and *Cyp3a25*, which mediate bile acid detoxification and whose expression is unregulated by Pxr and Car. Further in line with previous studies, HFD-intake decreased expression of *Cyp8b1*^25^, an enzyme of the alternative bile acid synthesis pathway, and increased expression of the steroid hormone synthesis genes *Hsd3b2*^25,30^, *Hsd3b3*^25,30^ and *Hsd17b11*. In summary, HFD-feeding decreased expression of cholesterol synthesis genes, and increased expression of hepatocellular cholesterol uptake, cholesterol-to-bile acid conversion, bile acid detoxification and excretion, and steroid hormone synthesis genes (Fig. 2c).

#### Fatty acid metabolism

DAVID and BiNGO/Cytoscape3.2 mapped 16 and 17 DEG functioning in FA metabolism (GO-ID: 6631) (Table S4), respectively. In addition, we found 8 unmapped DEG participating in FA metabolism (Table S4). As reported in previous studies of livers of HFD-exposed C57BL/6 mice, HFD-feeding stimulated expression the FA uptake gene *CD36*^23,25,30^, and the intracellular FA transport gene *Fabp2*^23–25^. In further alignment with previous studies, HFD-feeding increased expression of genes involved in FA β-and ω-oxidation: (i) *Ehhadh*^23^ and *Acaa1b*^23^ encoding enzymes of the peroxisomal β-oxidation pathway; (ii) *Cyp4a10*^24,26^ encoding an enzyme of the microsomal ω-oxidation pathway; (iii) *Cpt1*α^24,26^ encoding a transport protein for the import of long-chain FAs into mitochondria for β-oxidation; (iv) *Ppar*α^23,26,33^ encoding a nuclear receptor and key transcriptional activator of the β-and ω-oxidation pathways; (v) *Dio1*^30^ encoding an enzyme that activates the prohormone thyroxine (T4) to its active form tri-iodothyronine (T3), which in turn stimulates transcription of β-oxidation genes^34^; (vi) *Pex11a* encoding a membrane elongation factor required for peroxisome biogenesis; (vii) *Acot1*, *Acot2*, *Acot3*, *Acot4*, and *Acot11* encoding enzymes that release free FAs from acyl-CoA esters in peroxisomes for their subsequent import into mitochondria^35^. Consistent with earlier reports, HFD-feeding also upregulated several genes mediating triglyceride (TG) synthesis and storage: (i) *Srebf1*^23,25,30,33^ encoding a key transcription factor driving expression of FA and TG synthesis genes; (ii) *Scd1*^24,25^ encoding a FA desaturase and transcriptional target of *Srebf1*^36^ that desaturates stearic acid to oleic acid for its incorporation into TGs; (iii) *Thrsp* encoding an activator of FA and TG synthesis^37^, as well as transcriptional target of *Srebf1*^38^; iv) *G0s2*^24,30^ encoding an intracellular storage protein for TGs^39^. Moreover, we found putative transcription factor binding sites for Srebf1 in the promoters of *Scd1* and *Thrsp* (Table S12), supporting the notion that they are transcriptional targets of Srebf1. As reported earlier, HFD-feeding also led to gene expression changes decreasing FA synthesis: (i) downregulation of genes encoding enzymes of the FA synthesis pathway, namely *Acss2*^23,27^, *Elovl1*^23^ and *Elovl3*^24,25^; (ii) upregulation of *Erbb4* and *Nrg4*, encoding an epidermal growth factor receptor and its agonist, that shut off transcription of FA synthesis genes in liver^40^. In summary, HFD-induced transcriptional gene expression promoted uptake, intracellular transport and oxidation of FAs, decreased FA synthesis, and stimulated synthesis and storage of TGs (Fig. 2c).

#### Lipid localization

DAVID and BiNGO/Cytoscape3.2 mapped 12 and 11 DEG functioning in lipid localization (GO-ID: 10876) (Table S4), respectively. Furthermore, we identified 4 unmapped DEG modulating lipid localization (Table S4). HFD-feeding up-regulated *CD36*, encoding a cholesterol and FA uptake transporter, and *Fabp2*, encoding an intracellular FA transport protein. Consistent with previous studies, HFD-feeding downregulated genes mediating intracellular cholesterol transport, namely *Stard4*^27^ and *Cav1*^41^. Stard4 is a hypothesized intracellular cholesterol transport protein containing a cholesterol-binding STAR-related lipid transfer (START) domain^42^. Cav1 transfers newly synthesized cholesterol from the endoplasmic reticulum to plasma membrane invaginations called caveolae, where cholesterol helps anchoring plasma membrane proteins^43^. Further in line with previous studies, HFD-feeding up-regulated *Abcg2*^23,25^, *Abcg5*^23,25,27,44^, and *Abcg8*^23,25,44^. These genes encode membrane-associated ABC transporters mediating the biliary excretion of cholesterol and bile acids. HFD-feeding also led to previously reported gene expression changes altering reverse cholesterol transport, a process, whereby HDL particles move cholesterol in plasma from peripheral tissues to the liver: (i) up-regulation of *Abca1*^23,25^ encoding a membrane transport protein required for the hepatic formation of HDL particles^45^; (ii) up-regulation of *Apoa4*^23,24,27^ encoding a secreted protein that facilitates sequestration of cholesterol into HDL particles; (iii) down-regulation of *Pltp*^24,25,27^ encoding a secreted protein that facilitates loading of cholesterol onto HDL in peripheral tissues. Consistent with previous studies, HFD-feeding also upregulated genes encoding intracellular lipid storage proteins, namely *Cidea*^27,46^, *Cidec*^24,46^ and *G0s2*^24,30^. In summary, HFD-induced gene expression promoted cellular uptake of FAs and cholesterol, intracellular FA transport, intracellular TG storage, bile acid and cholesterol excretion, and reverse cholesterol transport (Fig. 2c).

#### Circadian rhythm

DAVID and BiNGO/Cytoscape3.2 mapped 6 DEG regulating the circadian rhythm (Table S4). In addition, we identified 3 unmapped DEG known to control the circadian rhythm (Table S4). Livers in the STD and HFD groups were collected at the same time of the day, thus excluding the time of collection as confounding factor. Metabolic processes are transcriptionally regulated as 24-hour oscillations of gene expression by clocks present in most peripheral tissues. The core of these clocks consists of a set of transcription factors encoded by the *Clock*, *Arntl*, *Period* (*Per*), and *Cryptochrome* (*Cry*) genes. Clock and Arntl form a heterodimer that activates transcription of the *Period* and *Cryptochrome* genes as well as other clock and metabolic genes. When Per and Cry proteins accumulate above a threshold level, they block Clock/Arntl transcriptional activity. This negative feedback occurs every 24 h resulting in rhythmic expression of core clock genes and metabolic genes. Consistent with previous studies of livers of HFD-fed C57BL/6 mice, HFD-feeding down regulated the core clock genes *Clock*^33^ and *Arntl*^24,25,33^, and up-regulated the core clock genes *Per2*^25,33^, and *Per3*^25^. HFD-feeding also altered expression of genes participating in accessory inhibitory feedback loops to the core clock, namely *Rev-Erb-*α (*Nr1d1*), *Usp2*, *Ciart* and *Prox1*. It down regulated *Rev-Erb-*α (*Nr1d1*) and up-regulated *Usp2*, *Ciart* and *Prox1*. While previous studies reported the downregulation of *Rev-Erb-*α (*Nr1d1*)^33^ and upregulation of *Usp2*^24^ in livers of HFD-fed C57BL/6 mice, the HFD-induced upregulation of *Ciart* and *Prox1* are novel observations. In summary, HFD-feeding altered transcriptional regulation of the core circadian clock and its accessory negative feedback loops (Fig. 2c).

### Expression profiling of lncRNA genes

We ranked hepatic expression of lncRNA genes based on average expression levels across all STD and HFD liver RNA pools (Table S5). 343 lncRNAs (182 lincRNAs, 151 antisense RNAs, 9 sense-intronic RNAs and 1 sense-overlaping RNA) were expressed at levels >1 CPM. The ten most abundant lncRNAs were Yam1, Malat1, Gm26870, RP23-181K4.2, B430119L08Rik, 1700020I14Rik, Neat1, Kcnq1ot1, Gm15564, and Brip1os.

### Co-expression analysis of lncRNA and protein-coding genes *in cis*

We conducted co-expression analyses of lncRNA genes with their nearest flanking or overlapping protein-coding genes. Only lncRNA and protein-coding genes with average expression levels across all samples >1 CPM were considered for analysis. We found 299 lncRNA/protein-coding gene pairs formed by 283 lncRNA genes and 281 protein-coding genes. In 53 lncRNA/protein-coding gene pairs formed by 52 lncRNA genes and 52 protein-coding genes, lncRNA and protein-coding genes were co-expressed, as evidenced by the Pearson’s correlation coefficient (critical *r* value: >0.73 or <−0.73 for one-tailed test, n = 6, *p* < 0.05; Table S6). Co-expressed lncRNA genes comprised lincRNA genes (32 or 61.5%), antisense RNA genes (19 or 36.5%) and sense-intronic RNA genes (1 or 2%). LncRNA and protein-coding gene expression correlated positively in 69.8% and negatively in 30.2% of all co-expressed lncRNA/protein-coding gene pairs (Table S6). LncRNA genes were located up- or downstream of their co-expressed protein-coding gene. Furthermore, lncRNA genes overlapped the protein-coding gene promoter and body in 58.5% and 41.5% of all co-expressed lncRNA/protein-coding gene pairs, respectively (Table S6). We found no overlap of co-expressed lncRNA genes with known mouse enhancers annotated in VISTA^47^, a comprehensive database for highly conserved human and mouse enhancers, suggesting those lncRNA genes may not be enhancer derived RNAs (eRNAs). LncRNA genes displayed most frequently lower expression levels than their co-expressed protein-coding genes (Table S6). Gene ontology classification by biological processes revealed that co-expressed protein-coding genes function in lipid metabolism (*Ap2a2*, *Crot*, *Hsd17b7*, *Pecr*, *Smpd3*), cell adhesion (*Col4a3*, *Hmcn1*, *Sorbs2*), cell division (*Cep83*, *Kmt2e*, *Mafg*, *Nacc2*, *Rassf1*, *Seh1l*, *Sfi1*, *Sin3a*, *Smpd3*, *St3gal5*), cell migration/movement (*Cep83*, *Sorbs2*), apoptosis (*Jade1*, *Sin3a*), proteolysis (*Adamts9*, *Hmces*, *Pcbp2*, *Serpinb6a*, *Ube4b*), endocytosis/exocytosis (*Als2*, *Apa2a*, *Dnm3*, *Rapgef4*), cellular transport of small molecules, lipids and proteins (*Als2*, *Ap2a2*, *Kif5b*, Mup3, *Scyl1*, *Slc1a2*, *Slc3a2*), signal transduction (*Als2*, *Dnm3*, *Pik3r2*, *Phb2*, *Rapgef4*, *Rgs16*, *Sin3a*, *Tulp3*), chromatin remodeling (*C130026I21Rik*, *Jade1*, *Nacc2*, *Kmt2e*, *Mettl4*), RNA processing (*Dhx15*, *Ints2*, *Mettl4*), and transcription (*Zbtb5*, *Irf2*, *Ints2*, *Jade1*, *Mrpl12*, *Mafg*, *Tulp3*, *Zfp148*, *Kmt2e*, *Zfp619*, *C130026I21Rik*) (Table S6).

### Diet-responsive lncRNA genes

EdgeR identified 14 differentially expressed lncRNA genes with average expression values across all livers >1 CPM (Tables 4 and S3). nCounter® technology confirmed differential expression of 11 examined lncRNA genes (Table 4). Expression differences could not be validated for 3 lncRNA genes due to unavailability of target-specific genomic nCounter® probes. Fold-change values determined by nCounter® technology were smaller than those determined by RNA-seq. This is not surprising because RNA-seq tends to overestimate fold-changes of very rare transcripts with low read counts such as lncRNAs.

**Table 4:** Diet-induced differential expression of lncRNA genes.

Gene symbol	edgeR FC (H/S)	edgeR FDR	nCounter FC (H/S)	nCounter *p*-Value	nCounter targeted transcript isoforms
A330076H08Rik	0.02	1.9E-03	0.74	3.29E-01	ENSMUST00000181804
			0.45	1.17E-02	ENSMUST00000181187
A530040E14Rik	0.06	6.0E-02	0.39	1.95E-02	ENSMUST00000161666
					ENSMUST00000162453
			0.29	3.47E-03	ENSMUST00000162353
					ENSMUST00000093501
					ENSMUST00000161150
AY512915	0.01	2.2E-03	0.39	1.81E-02	ENSMUST00000089303
			0.67	1.78E-01	ENSMUST00000159751
			0.54	3.85E-02	ENSMUST00000162315
			0.55	9.59E-02	all transcript isoforms
Dlx6os1	0.01	1.8E-04	0.39	2.42E-02	ENSMUST00000159827
Gm12002	0.003	3.8E-03	0.38	4.16E-03	ENSMUST00000153794
			0.72	7.83E-03	ENSMUST00000174550
Gm13582	0.02	2.1E-04	0.50	5.06E-02	all transcript isoforms
Gm16025	0.06	3.95E-02	0.56	1.67E-02	ENSMUST00000161282
			0.81	2.11E-02	all transcript isoforms
Gm16028	0.06	1.5E-02	0.20	7.95E-03	all transcript isoforms
Gm20631	0.06	9.1E-03	0.40	7.51E-03	ENSMUST00000177239
			0.39	2.00E-02	ENSMUST00000177268
			0.48	1.34E-02	all transcript isoforms
Gm21846	0.04	1.7E-02	0.52	6.53E-03	All transcript isoforms
Wt1os	0.003	3.1E-05	0.67	1.96E-01	ENSMUST00000172701
					ENSMUST00000174870
			0.42	1.97E-02	ENSMUST00000135153
Gm26870	0.19	1.5E-08	n.d.	n.d.	n.d.
Gm11399	0.07	6.4E-03	n.d.	n.d.	n.d.
D230017M19Rik	0.1	2.7E-03	n.d.	n.d.	n.d.

Differential expression of lncRNA genes was validated by nCounter® technology in individual livers (n = 6/diet). nCounter® probes targeted all or specific transcript isoforms of a lncRNA gene. Specifically targeted transcript isoforms are denoted by their Ensembl transcript IDs. nCounter® fold-change values were not determined (n.d.) for *Gm26870*, *Gm11399*, *D230017M19Rik* due to lack of specific nCounter® probes. FC (H/S) refers to the fold-change (FC) in expression in HFD livers (H) relative to STD livers (S). nCounter® *p*-values were calculated by two-sided *t* test. edgeR FDR refers to the False Discovery Rate-adjusted *p*-value calculated by edgeR.

### Diet-responsiveness of co-expressed lncRNA/protein-coding gene pairs

Only 4 co-expressed lncRNA/protein-coding gene pairs were responsive to the diet as reflected by the differential expression of both, lncRNA gene and coding gene (*Gm26870*/*Gm10717*, *Gm11399*/*Sfi1*, *A530040E14Rik*/*C130026I21Rik*, *Gm16025*/*C130026I21Rik*) (Table S7). Co-expressed diet-responsive protein-coding genes had functions in cell division (*Sfi1*), possibly in transcription and chromatin remodeling (*C130026I21Rik*) or had unknown function (*Gm10717*). Hence, none of the co-expressed diet-responsive protein-coding genes functioned in overrepresented biological processes.

### Genome-wide assessment of DNA methylation by 5meDIP-seq

We studied diet-induced DNA methylation of coding genes as a potential epigenetic endpoint of lncRNA/protein-coding gene interactions. To this end, we conducted 5meDIP-seq of the same STD (n = 3) and HFD (n = 3) liver pools used for RNA-seq. Methylated DNA was immunoprecipitated with a mouse monoclonal antibody raised against 5-methylcytosine. Real-time PCR amplification of known methylated sites in the *Zc3h13* and *Grik3* genes and one unmethylated site in the untranscribed region *Untr6* confirmed efficient and specific pulldown of 5-methylcytosine DNA (Fig. S2). Sequencing libraries were prepared from the DNA immunoprecpitates and single-end sequenced. The reads were aligned to the mouse genome by the Burrows Wheeler Aligner^48^. On average, 12996741 pass-filter reads (STD: 14028303; HFD: 11965179) aligned with no more than 2 mismatches to a unique genomic site and were not exact duplicates resulting from the PCR amplification of the sequencing library (Table 5). Of these, 10 million reads were fed into the SICER algorithm^49^ for peak detection relative to reads obtained from input (not immunoprecipitated) DNA pooled from all the samples. Overlapping peaks across all samples were grouped into “methylated regions”, whose margins were defined by the start coordinate of the most upstream peak region and the end coordinate of the most downstream peak region across all samples. When a peak was detected only in one sample, that peak defined the methylated region. Between 59000 and 97000 methylated regions were detected in each of the six liver pools (Table S8), for a total of 154664 detected methylated regions. 36383 methylated regions were shared by all six liver pools. Methylated regions were distributed across intergenic sequences (65105 or 42%) and genes (89559 or 58%) within boundaries of 10 kb from the genes’ first and last exon (Fig. S3a). These intragenic methylated regions were located in protein-coding genes, non-coding genes and pseudogenes, mapping foremost to the gene body only (59562 or 67%; Fig. S3b). Less frequently, they mapped to the 10 kb upstream region only (9111 or 10%), the 10 kb downstream region only (8036 or 9%), or combinations of the 10 kb upstream region, gene body, and 10 kb downstream region of the same gene, adjacent genes or overlapping genes (12850 or 14%) (Fig. S3b). Finally, 1690 methylated regions mapped to annotated CpG Islands (CGIs), most of which were intragenic (Fig. S3c).

**Table 5:** 5meDIP-seq alignment statistics.

Sample	Number of reads	Number of alignments	Number of unique alignments	Number of unique alignments without duplicate reads
HFD A	33550731	31488970	15502775	10051886
HFD B	33370167	31294850	16434763	10894779
HFD C	36995355	34708215	19888168	14948873
STD A	37931887	35475014	19716455	13654052
STD B	39045552	36407892	20167366	14222042
STD C	36576656	34298870	19934085	14208816
Average	36245058	33945635	18607269	12996741
Average HFD (n = 3 pools)	34638751	32497345	17275235	11965179
Average STD (n = 3 pools)	37851365	35393925	19939302	14028303

5-methylcytosine DNA immunoprecipitates (n = 3 pools of 2 livers/diet) were single-end sequenced. The number of alignments is the number of reads that aligned with no more than 2 mismatches to the mouse genome. The number of unique alignments refers to the number of reads that aligned to a unique site of the mouse genome with no more than 2 mismatches. The number of unique alignments without duplicate reads is the number of reads that aligned to a unique site of the mouse genome with less than 2 mismatches and were not exact duplicates produced by PCR amplification of the sequencing libraries. Of these, exactly 10 million reads were used for methylation analysis.

### Analysis of diet-induced DNA methylation by 5meDIP-seq

**Figure 3:**
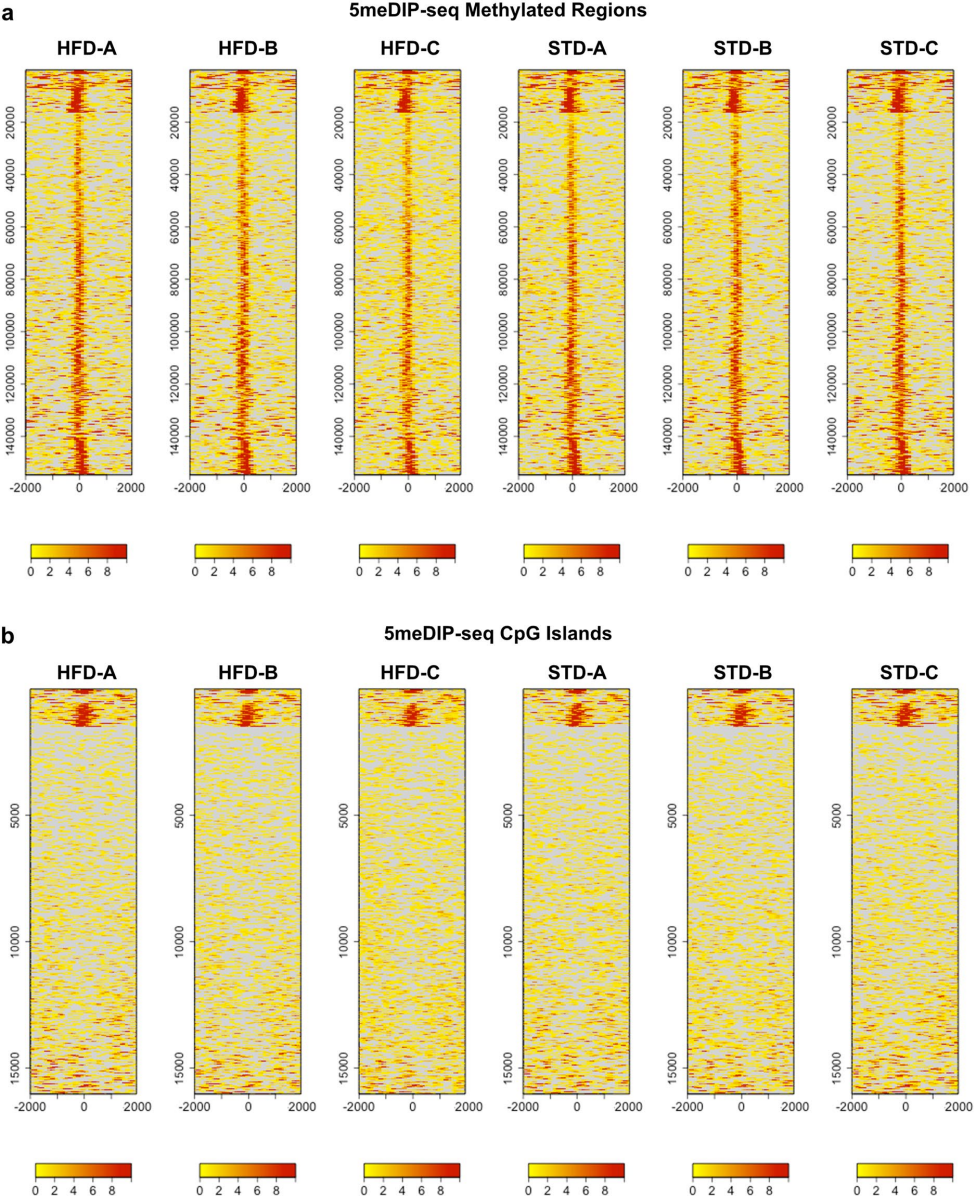
Heatmap of DNA methylation in STD and HFD livers determined by 5meDIP-seq. (**a**) K-means clustering of 154664 methylated regions (y-axis) by peak signal (fragment counts) from −2000 bp to +2000 bp relative to midpoint (x-axis). (**b**) K-means clustering of 16026 CpG islands (y-axis) annotated in the UCSC genome browser by peak signal (fragment counts) from −2000 bp to +2000 bp relative to midpoint (x-axis). The color code in (**a** and **b**) reflects the number of fragments at the specified position. All heatmaps were generated using seqplots and a default cluster size of 5.

We assessed differential DNA methylation by comparing the average extended read (fragment) counts of corresponding methylated regions in STD and HFD livers by the *t* test, followed by correcting the *p*-values for multiple testing by the family-wise error rate (FWER) and the less stringent false discovery rate (FDR). While we found a number of methylated regions with non-adjusted *p*-values < 0.05, none of them showed significant *p*-values < 0.05 after FWER correction by the Bonferroni, Holm, or Hochberg method or FDR correction by the Benjamini/Hochberg or Benjamini/Yekutieli methods (Table S9; Fig. 3). Moreover, methylated regions with non-adjusted *p*-values < 0.05 showed very weak peaks of less than 10 or 20 fragment counts, which are likely to be more variable. We also tested for differential methylation by evaluating differences in read abundance between corresponding methylated regions using DESeq2 (see Methods). This program was originally developed for the analysis of RNA-seq data, but DESeq2 is now also used for the differential analysis of ChIP-seq peaks. For example it is used internally in the DiffBind program (https://bioconductor.org/packages/release/bioc/html/DiffBind.html) as well as in the ChIP-seq pipelines of Genomatix (https://www.genomatix.de/online_help/help_regionminer/ChIPSeqWorkflow.html) and Homer (http://homer.ucsd.edu/homer/ngs/peaksReplicates.html). DESeq2 confirmed the absence of differentially methylated regions with significant FDR-adjusted *p*-values < 0.1 (Table S9). Therefore, 5meDIP-seq revealed only very slight differences in DNA methylation between STD and HFD livers and these were not significant with the statistical methods applied.

### Analysis of diet-induced DNA methylation by targeted BS-seq

To validate the assessment of differential DNA methylation by 5meDIP-seq, we bisulfite-sequenced 10 methylated regions mapping to the *Apoa4*, *Cidec*, *Ddit4*, *Dio2*, *Ehhadh*, *Lpin2*, *Mvd*, *Per2*, *Tank*, and *Wwc1* genes (Table S10). These methylated regions were selected for analysis because some of them were potentially weakly differentially methylated by 5meDIP-seq (non-adjusted *p*-values < 0.05; Table S9) and the respective protein-coding genes (*Apoa4*, *Cidec*, *Mvd*, *Per2*) were differentially expressed and functioned in one of the overrepresented pathways. BS-seq was performed in the same liver pools (n = 3/diet) used for RNA-seq and 5meDIP-seq. Each methylated region contained between 4 and 16 CpG sites (Table S10). For BS-seq, the genomic DNA was first treated with bisulfite to convert non-methylated cytosine to uracil. Methylated regions were then PCR-amplified. The PCR products were concatamerized, fragmented by sonication and single-end sequenced. Reads were aligned to the mouse reference genome using the Bismark alignment program (v 0.7.7)^50^. Between 5–6 million reads were analyzed per sample. The alignment coverage for each CpG site varied moderately but was much over 1000 in all liver pools (Table S10). Most methylated regions showed strong CpG site methylation in all liver pools (Table S10). STD and HFD liver pools displayed no differences in CpG site methylation in 9 of the 10 sequenced methylated regions (Table S10), consistent with lack of significant adjusted *p*-values for differences in DNA methylation by 5meDIP-seq. However, one methylated region in the *Wwc1* gene showed a trend for decreased CpG site methylation in HFD livers. Very low non-CpG site methylation (<1%) was observed in several methylated regions (Table S11). This is expected, as these C-residues are usually not methylated in mammalian cells. In summary, BS-seq confirmed the absence of diet-induced DNA methylation in 9 of 10 examined methylated regions.

## Discussion

Exposure of C57BL6/J mice to a 12-week Western-style HFD altered hepatic expression of 395 protein-coding genes, which were statistically overrepresented for functions in steroid and cholesterol metabolism, FA metabolism, lipid localization, and circadian rhythm. Deconvolution analysis of cell-type specific marker gene expression revealed no substantial differences in cell type composition between STD and HFD livers, suggesting that differences in gene expression were not primarily due to differences in cell type composition.

The majority of gene expression changes were enriched for functions in steroid and cholesterol metabolism. This is expected from the supplementation of cholesterol in the HFD. HFD-feeding repressed expression of numerous cholesterol synthesis genes and increased expression of genes mediating cholesterol uptake, cholesterol-to-bile acid conversion, bile acid detoxification and biliary excretion of cholesterol and bile acids. HFD-feeding also led to gene expression changes enhancing reverse cholesterol transport. The repression of cholesterol synthesis genes can be explained by hepatocyte accumulation of cholesterol and subsequent feedback inhibition of Srebf1 and Srebf2, two key transcription factors driving expression of cholesterol synthesis genes. In support of this notion, our bioinformatics search for transcription factor binding sites and peaks from previous ChIP-seq experiments indicated the presence of putative Srebf1 and Srebf2 binding sites in the promoters of most differentially expressed cholesterol synthesis genes. Repression of cholesterol synthesis genes may also be explained by ligand-activation of the nuclear receptor Lxr-α by cholesterol metabolites, so-called oxysterols. In turn, Lxr-α represses transcription of the cholesterol synthesis genes *Cyp51*^51^, *Fdft1*^51^, *Hmgcs*^52^, *Hmgcr*^52^. Furthermore, Lxr-α is known to drive transcription of the cholesterol efflux transporter gene *Abca1*^53^ and the cholesterol and bile acid excretion genes *Abcg5*^54^ and *Abcg8*^54^. Hence, its activation may also account for hepatic up-regulation of cholesterol efflux and excretion genes in response to chronic cholesterol feeding.

The second and third most frequent diet-induced expression changes regarded genes functioning in FA metabolism and lipid localization, respectively. Genes mediating the uptake, intracellular transport and β-oxidation of FAs, as well as the synthesis and storage of TGs were upregulated while FA synthesis genes were downregulated. Moreover, genes mediating cellular uptake of cholesterol, biliary excretion of cholesterol and bile acids, and formation of HDL particles for reverse cholesterol transport were upregulated.

Finally the fourth most frequent diet-induced expression changes concerned genes regulating the circadian clock. HFD altered expression of core clock genes and genes participating in negative feedback loops to the core clock. The manner whereby HFD-feeding alters the liver core clock, in terms of amplitude or phase of circadian expression of clock genes and clock-controlled metabolic genes, remains to be clarified. Diet-induced dysregulation of the liver circadian clock may contribute to diet-induced expression changes of lipid metabolism genes. Indeed, a HFD was found to increase the circadian mRNA amplitudes of *Ppar*α and *Ppar*γ^33^ or shift the phase of the circadian mRNA oscillations of *Srebf1*^33^. In addition, it is conceivable that HFD feeding might have altered circadian behavior and feeding patterns of mice to impact circadian gene expression in liver.

RNA-seq detected hepatic expression of 343 lncRNA genes expressed at levels >1 CPM. Of these, 14 lncRNA genes of yet unknown function were differentially expressed in response to HFD feeding. Co-expression analyses suggested *cis*-regulatory interactions for 53 lncRNA/protein-coding gene pairs at an expression cut-off >1 CPM. Of the above 14 diet-responsive lncRNA genes only 4 were co-expressed with adjacent or overlapping differentially expressed protein-coding genes none of which had biological functions in overrepresented pathways. Hence, in mouse liver, transcription of lncRNA genes is not linked to transcription of adjacent or overlapping lipid metabolism, lipid localization and circadian clock genes in response to HFD feeding.

This modest number of diet-responsive co-expressed lncRNA/protein-coding gene pairs may be explained by the low number or fraction of differentially expressed potein-coding genes (100 of 395; 25.3%) that are flanked or overlapped by annotated lncRNA genes. In addition, the vast majority of these flanking or overlapping lncRNA genes (132 of 144; 92%) were not expressed at all or expressed at extremely low levels below the expression cut-off of 1 CPM. And only 12 lncRNA genes were expressed above this threshold and available for co-expression analysis. It is possible that non-expressed or extremely rare lncRNAs are transcribed during liver development or in a subset of specific cell types^55^, and thus escape detection. More sensitive and complex RNA-seq technologies and data analyses to detect diet-responsive lncRNA/protein-coding gene interactions may clarify these considerations. These include RNA-seq of specific cell type populations; RNA-seq of the nuclear or the chromatin-associated RNA fractions; computational discovery of non-annotated lncRNAs through de novo re-assembly of the mouse hepatic transcriptome; and analysis of alternative non-linear lncRNA/protein-coding gene interactions.

5meDIP-seq revealed no differential DNA methylation of differentially expressed genes. Moreover, targeted BS-seq confirmed the vast absence of diet-induced differential DNA methylation in 9 of 10 examined methylated gene regions. These results contrast with previous studies reporting differentially methylated genes in rodent^56–58^ and human^59,60^ fatty livers. The reasons for these contrasting study outcomes remain to be clarified. They may relate to differences in the micro- or macronutrient composition of the diets, as well as to diet-induced differences in hepatic cell type composition, species-specific genetic background, or genome technologies used for profiling DNA methylation. We cannot exclude that diet-induced DNA methylation occurs in a subset of cell types and might have escaped detection. Therefore, future studies should readdress the occurrence of diet-induced DNA methylation in specific cell type populations. Ideally this would be done at single base resolution by whole genome BS-seq, which would likely provide more detailed information than 5meDIP-seq. However, for mammalian systems, BS-seq currently requires a lot more sequencing and data analysis to make it a feasible and affordable alternative to 5meDIP-seq.

## Methods

### Ethics Statement

Care of mice and animal procedures were carried out with the approval of the Institutional Animal Care and Use Committee of the University of Miami and in accordance with the relevant guidelines and regulations.

## Animals

Animals used throughout the study were males of the C57BL6/J mouse strain (RRID:IMSR_JAX:000664, Jackson Laboratories, Bar Harbor, Maine). 6-week old males were group-housed in cages of 4 animals prior to the start of the study at the age of 8 weeks when they were single housed with environmental enrichment until the end of the study. All mice were housed in a pathogen-free barrier animal facility and kept in a temperature- (22 ± 0.5 °C) and humidity- (50%) controlled animal room on a 12-hour light/dark schedule with light on at 0700 h and free access to food and water.

### Diets

C57BL/6J male mice were fed a STD with a metabolizable energy density of 3.1 kcal/g derived from 24% kcal protein, 60% kcal carbohydrates, and 16% kcal fat (2020x, Teklad Diets, Harlan, Madison, WI, USA). At the age of 8 weeks, a subset of the animals was subjected to a HFD with a metabolizable energy density of 4.7 kcal/g derived from 17% kcal protein, 43% kcal carbohydrates, and 41% kcal fat, containing 0.21% (w/w) Cholesterol for 12 weeks (Western Diet, Research Diets Inc., New Brunswick, NJ, USA).

### Tissue harvesting

At the end of the 12-week STD or HFD, non-fasted animals were sacrificed in the morning between the hours of 0900 and 1200. Livers were immediately collected, frozen on dry ice and stored at −80 C until further use.

### RNA extractions

Equal amounts of frozen liver samples (30 mg) were homogenized in lysis buffer at room temperature for 4 minutes using a ball mixer mill (Retsch, Germany). Total RNA was immediately column-purified and DNAse digested using the RNeasy kit (Qiagen, USA). The Agilent 2100 Bioanalyzer system (Agilent, California, USA) yielded RNA integrity numbers between 7.4 and 9.5, thus confirming high-level integrity of all liver RNA extracts.

### RNA sequencing

3 pools of 2 mouse livers in the STD and HFD groups were RNA sequenced. A directional RNA-seq library was generated for each RNA pool using the ScriptSeq™ v2 RNA-Seq Library Preparation Kit (Epicentre, Wisconsin, USA). RNA-seq libraries were paired-end sequenced on Illumina’s HiSeq2000 sequencer. The read length was 99 bp. Pass-filter read-pairs were aligned to the mouse reference mm10 genome using the STAR aligner^14^. Only read-pairs aligning to a unique site of the genome with less than 2 mismatches were used for analyses. The aligned read-pairs were run through HTSeq (RRID:SCR_005514; http://www-huber.embl.de/users/anders/HTSeq/) for determination of read-pair counts by genes under consideration of strand-specificity against the GENCODE reference gtf file vM4 for the mouse mm10 genome^15^. Expression of a gene was normalized by dividing the number of read-pairs mapped to its exons by the number of all read-pairs mapped to the genome. The resulting value was expressed in counts per million (CPM). Expression of individual alternative transcript isoforms was not assessed. After genes had been quantified by HTSeq, the data was run through the differential expression calculator edgeR (RRID:SCR_012802; http://www.bioconductor.org/packages/release/bioc/html/edgeR.html)^16^. EdgeR uses an overdispersed Poisson model to account for both biological and technical variability, as well as Empirical Bayes methods to moderate the degree of overdispersion across transcripts, improving the reliability of inference. A False discovery rate (FDR) threshold <0.05 prioritized genes of interest. For two-way clustering, dendrogram and heatmap generation, the default parameters of the heatmap.2 function within the R package gplots v2.16.0 (http://cran.rproject.org/web/packages/gplots/index.html) was used. Gene ontology and pathway analyses were conducted using DAVID (Database for Annotation Visualization and Integrated Discovery, RRID:SCR_003033; http://david.abcc.ncifcrf.gov/)^21^ and the BiNGO (BiNGO: A Biological Networks Gene Ontology tool, RRID:SCR_005736; http://www.psb.ugent.be/cbd/papers/BiNGO/Home.html) add-on application to Cytoscape 3.2 (RRID:SCR_003032; http://cytoscape.org)^22^. Cell type frequencies in liver were calculated in R using the Digital Sorting Algorithm (DSA)^18^ and meanProfile algorithm within the CellMix1.6 package^19^ by deconvolving expression of the following cell-type specific marker genes: a) hepatocytes: *Baat*, *Hpx*, *Apoa5*, *Itih1*, *F12*; b) Kupffer cells: *Mrc1*, *Fcgrt*, *Vsig4*; c) sinusoidal endothelial cells: *Clec4g*, *Stab2*, *Flt1*.

### nCounter® digital gene expression measurement

The nCounter® analysis system (NanoString Technologies, Seattle, WA, USA) is a liquid phase direct hybridization technology for detection of target RNAs using color-coded barcodes attached to target-specific probes and allowing multiplexed expression analysis of target RNAs^17^. Assayed mouse livers were the same ones used for RNA-seq. Total RNA was extracted from individual mouse livers, analyzed on an Agilent BioAnalyzer and showed RNA integrity numbers ranging from 7.4 to 9.5. Total RNA was assayed following the manufacturers standard gene expression assay protocol. 100 ng and 1 μg of total RNA were used for determination of mRNA and lncRNA levels, respectively. 12 housekeeping genes were assayed simultaneously for normalizing transcript counts. Transcript counts were calculated using the nSolver Analysis Software 1.1 (RRID:SCR_003420; http://www.nanostring.com/products/nSolver).

### 5-methylcytosine DNA immunoprecipitation sequencing

5meDIP-seq was conducted by Active Motif (Carlsbad, CA, USA; RRID:SCR_013589). 3 pools of 2 mouse livers in the STD and HFD groups were subjected to 5meDIP-seq. Liver pools were the same used for RNA-seq. DNA was extracted from the liver pieces by digestion with proteinase K, extractions with phenol-chloroform (2:1) and chloroform, followed by ethanol precipitation in the presence of 2 M NH4Ac. The ethanol precipitated DNA was resuspended in TE (Tris 10 mM, pH 8, EDTA 1 mM) and digested with RNAse A, followed by organic extraction and ethanol precipitation as described above. DNA was sonicated using a Misonix Sonicator 300 equipped with a microtop. Illumina PE adaptors^61^ were added to 5 μg of sonicated DNA essentially following the protocol described by Lister *et al*.^62^. Adapter-ligated DNA was immunoprecipitated using a mouse monoclonal antibody against 5-methylcytosine (5meC) (Clone 33D3, Active Motif, Carlsbad, CA, USA) following the protocol outlined in the Active Motif MeDIP kit (Active Motif, Catalog No. 55009). This antibody shows no cross-reactivity with 5-hydroxymethylcytosine as determined by Active Motif. Specific pulldown of 5meC DNA was confirmed by real-time PCR using primers to known methylated sites in the *Zc3h13* and *Grik3* genes and to one unmethylated site in an untranscribed region called “*Untr6*”. The primer sequences were as follows: Grik3-A, ATGGTCTTGGGGGAACAGTA; Grik3-B, CAATTCCTCGGGCTCATAAA; Zc3h13-A, AGGAAGAAGCCCGCAGTTAT; Zc3h13-B, CAGGCAGTTCTGGACCTCTC; Untr6-A, AATACCAATGTCCACCCTCTG; Untr6-B CAACATCCACACGTCCAGTG. Deep sequencing libraries were generated from the DNA immunoprecipitates and input DNA by the standard consecutive enzymatic steps of end-polishing, dA-addition, and adaptor ligation. After a final PCR amplification step using barcoded primers, the resulting DNA libraries were quantified and sequenced on Illumina’s Hi-Seq2000. Pass-filter 50-nt sequence reads were mapped to the mouse mm9 reference genome using the Burrows Wheeler Aligner (BWA, RRID:SCR_010910; http://bio-bwa.sourceforge.net/) with default settings. Only reads that aligned with no more than 2 mismatches, mapped to a unique genomic site, and were not exact duplicates and thus the result of the PCR library amplification during library production were used for subsequent analyses. Exactly 10 million sequence reads were used for further analyses. Since the sequence reads represented the 5′-ends of the IP-fragments, the reads were extended *in silico* using proprietary software from Active Motif at their 3′-ends to a length of 150 bp to match the fragment length of the library. The genome was divided into 32-nt bins and the number of fragments in each bin was determined. The resulting metric (“fragment density”) was used for the peak metrics in Table S9 (columns G-L, Count Avg Val; and columns T-Y, Count Peak Val) in the Supplemental data as well as for the heatmaps shown in Fig. 3. SICER (RRID:SCR_010843; http://home.gwu.edu/~wpeng/Software.htm)^49^ detected local enrichments in aligned reads (“peaks”) in individual 5meDIP samples relative to input control DNA pooled from all samples as a reference at a significance threshold of FDR 1E-10, window size of 200 bp, gap size of 0 bp, and fragment size of 150 bp. To account for differences in the locations and lengths of the peaks, overlapping peaks were grouped into methylated regions, which were defined by the start coordinate of the most upstream peak and the end coordinate of the most downstream peak across all samples, using a proprietary script by Active Motif. In locations, where only one sample had a peak, that peak defined the methylated region. After defining the methylated regions, their nearest or overlapping CpG islands, promoters and genes were determined. Differences in DNA methylation between STD and HFD groups were determined by comparing average fragment counts of corresponding methylated regions by *t* test. P-values were corrected for multiple testing by the family-wise error rate (FWER) using the Bonferroni, Holm, or Hochberg method and by the false discovery rate (FDR) using the Benjamini/Hochberg or Benjamini/Yekutieli methods. Differences in DNA methylation between STD and HFD groups were also determined using the DESeq2 software^63^. Even though this program was originally developed for the differential analysis of RNA-seq data, it is now also used for the differential analysis of ChIP-seq data and related assays that involve peak finding: the DiffBind program (https://bioconductor.org/packages/release/bioc/html/DiffBind.html) uses the DESeq2 algorithm internally, and the ChIP-Seq pipelines of Genomatix (https://www.genomatix.de/online_help/help_regionminer/ChIPSeqWorkflow.html) and Homer (http://homer.ucsd.edu/homer/ngs/peaksReplicates.html) use DESeq2. The first step of this differential analysis extracts the read counts for all SICER-defined methylated regions (see above) from the unnormalized BAM files of the 6 samples. PCR duplicates and multi-mapping reads are excluded from the counts as in our standard analysis described above. DESeq2 then normalizes the counts and calculates *p*-values and adjusted *p*-values (FDR). The default threshold for the identification of significantly differential regions is FDR = 0.1 For the heatmaps shown in Fig. 3, the fragment densities across the 154664 methylated regions and at 16026 CpG islands were clustered by the K-means of their fragment counts from −2000 bp to +2000 bp relative to midpoint. K-means clusters and heatmaps of methylated regions and CpG islands were generated using the Bioconductor R package “seqplots” (http://przemol.github.io/seqplots) and a cluster default size of 5.

### Bisulfite sequencing

BS-seq was carried out by Active Motif (Carlsbad, CA, USA; RRID:SCR_013589). BS-seq determined CpG site methylation of 10 selected methylated regions in the same liver pools used for RNA-seq and 5meDIP-seq. PCR primers to the target regions were designed with the MethPrimer program (RRID:SCR_010269; http://www.urogene.org/cgi-bin/methprimer/methprimer.cgi) to the plus strand^64^. Genomic DNA was bisulfite-converted using the EZ DNA Methylation-Gold kit (Zymo Research, Irvine, CA, USA, D5005). Target regions were amplified by PCR from the bisulfite-treated DNA using Invitrogen Supermix (ThermoFisher Scientific, Waltham, MA, USA, 10572–014). Equal amounts of the 10 PCR products were concatemerized with T4 DNA ligase (New England Biolabs, Ipswich, MA, USA, M0202), sonicated to an average fragment length of 150–300 bp, processed into standard, barcoded Illumina sequencing libraries and single-end sequenced on Illuminas HiSeq2500 sequencer. 50-nt reads were analyzed using the Bismark alignment program (v 0.7.7) (RRID:SCR_005604; http://www.bioinformatics.babraham.ac.uk/projects/bismark/)^50^. A set of chromosomes in the mouse reference genome mm9 containing the target regions was used as reference sequence. Between 5–6 million pass-filter reads were analyzed per sample. Differences in CpG site methylation percentages were assessed by two-tailed, unpaired *t* test and two-tailed Mann-Whitney test using Prism 5 (GraphPad Software Inc., La Jolla, CA, USA). A *p*-value < 0.05 was considered significant.

### Search for putative Srebf1 and Srebf2 transcription factor binding sites

We scanned the promoters of differentially expressed genes mediating cholesterol, fatty acid and triglyceride synthesis for Srebf11 and Srebf2 binding sites using the Eukaryotic Promoter Database (EPD; https://epd.vital-it.ch/)^28^. The EPD contains an annotated collection of eukaryotic POL II promoters, for which the transcription start site has been determined experimentally. It provides a search tool for transcription factor motifs contained in the JASPAR Core 2018 Vertebrates library. From the application’s main website we input a single mouse gene and then selected Srebf1 and Srebf2 in the Search Motif Tool to scan for putative binding sites −5000 bp to +1000 bp from the transcription start site (TSS) and at a cutoff *p*-value of 0.001. The search engine returned the 5′-end nucleotide position of the hits relative to the TSS with negative values indicating a position upstream of the TSS and positive values indicating a position downstream of the TSS. We then downloaded the promoter nucleotide sequence using the EPD Sequence Retrieval Tool and manually retrieved the binding motifs shown in Table S12, assuming a binding motif length of 10 nucleotides. Furthermore, to obtain experimental evidence for the presence of transcription factor binding sites, we studied the locations of peaks from previous ChIP-seq experiments in the Gene Transcription Regulation Database (GTRD; http://gtrd.biouml.org)^29^. GTRD is a database of transcription factor binding sites identified by ChIP-seq experiments for human and mouse. In GTRD, raw ChIP-seq data obtained from ENCODE and SRA were aligned and peaks called by different peak callers (MACS, SISSRs, GEM and PICS). We retrieved ChIP-seq peaks for mouse Srebf1 by going to the Advanced Search subsection called “Binding sites near the specified gene” and manually inputting the organism, gene symbol and the transcription factor of interest (1.2.6.3.1 Srebf1). ChIP-seq data for mouse Srebf2 was not available in GTRD. The available options for cell line and treatment were left at “any”. We changed the data set tab to the peak caller of interest (MACS, SISSRs, GEM or PICS). The maximum gene distance was set to 5000. The browser was selected as output type to visually inspect the location of the peaks relative to promoters and gene body regions. By clicking on the individual bars representing the peaks, we retrieved the chromosomal coordinates in the mouse mm10 (GRCm38) genome as well as the tissue and cell type for that peak. This information was extracted for all the peaks. Peaks that are bolded in Table S12 relate to the liver sample.

### Availability of supporting data

The BAM files supporting the RNA-seq results are available in the Sequence Read Archive (SRA) (http://www.ncbi.nlm.nih.gov/sra) under the accession number SRP070184. The FASTQ files supporting the 5-meDIP-seq results are available in the SRA under the accession number SRP070617. The FASTQ files supporting the BS-seq results are available in the SRA under the accession number SRP071182. Please note for all supporting data: HFD A-C are referred to as Obese A-C and STD A-C are referred to as Lean A-C.

## Electronic supplementary material

Supplementary Information

Supplementary Dataset 1

Supplementary Dataset 2

Supplementary Dataset 3

Supplementary Dataset 4

Supplementary Dataset 5

Supplementary Dataset 6

Supplementary Dataset 7

Supplementary Dataset 9

Supplementary Dataset 10

Supplementary Dataset 11

Supplementary Dataset 12
