# Supplementary material for: Analysis of diet-induced differential methylation, expression, and interactions of lncRNA and protein-coding genes in mouse liver
Source: Sci Rep. 2018 Aug 1;8:11537. doi: 10.1038/s41598-018-29993-4 (PMC6070528; doi:10.1038/s41598-018-29993-4)
Supplement: Supplementary file 1 — Supplementary Information [file 41598_2018_29993_MOESM1_ESM.pdf]

Supplementary Information

Analysis of diet-induced differential methylation, expression, and interactions of lncRNA and protein-coding genes in mouse liver

Jose P Silva[1,*], Derek van Booven[2]

[1] Department of Psychiatry and Behavioral Sciences and [2] John P Hussman Institute for Human Genomics, Miller School of Medicine, University of Miami, Miami, FL 33136, USA

[*] Correspondence: jossil68@gmail.com

Contents

Supplementary Figures

- **Figure S1**
- **Figure S2**
- **Figure S3**

Supplementary Tables

- **Table S1. Gene expression list**
- **Table S2. Differentially expressed genes and their flanking/overlapping lncRNA genes**
- **Table S3. Validation of edgeR-calculated gene expression changes by nCounter® technology**
- **Table S4. Differentially expressed genes in overrepresented biological processes**
- **Table S5. Expression ranking of lncRNA genes**
- **Table S6. Co-expression of lncRNA genes with their nearest protein-coding genes**
- **Table S7. Diet-responsive co-expression of lncRNA and protein-coding genes**
- **Table S8. Methylated region statistics**
- **Table S9. Methylated regions**
- **Table S10. CpG methylation**
- **Table S11. All C methylation**
- **Table S12. Putative Srebf1 and Srebf2 transcription factor binding sites**

Supplementary Datasets

- **Supplementary Dataset 1 to Table S1**
- **Supplementary Dataset 2 to Table S2**
- **Supplementary Dataset 3 to Table S3**
- **Supplementary Dataset 4 to Table S4**
- **Supplementary Dataset 5 to Table S5**
- **Supplementary Dataset 6 to Table S6**
- **Supplementary Dataset 7 to Table S7**
- **Supplementary Dataset 9 to Table S9**
- **Supplementary Dataset 10 to Table S10**
- **Supplementary Dataset 11 to Table S11**
- **Supplementary Dataset 12 to Table S12**

References to Supplementary Information

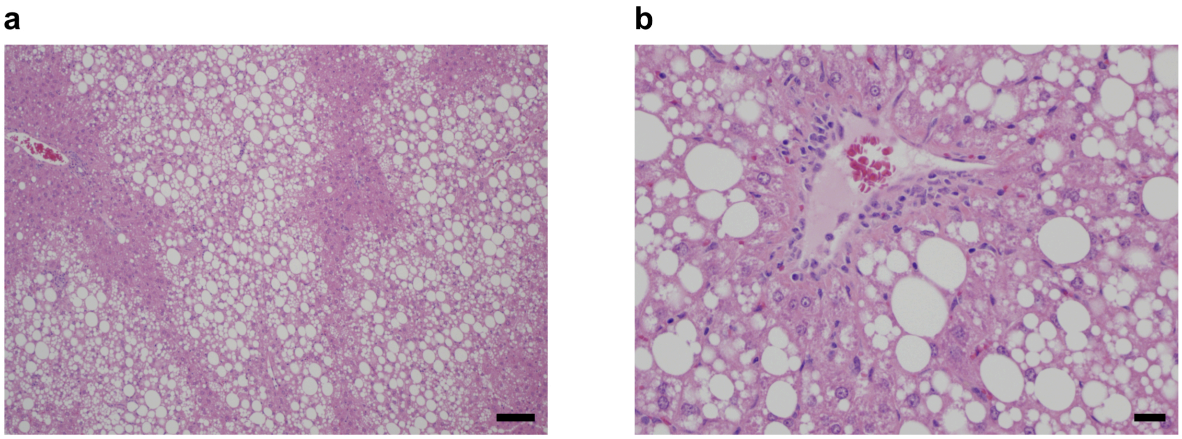

Figure S1. Hematoxylin and Eosin (HE) staining of livers shows a centrolobular micro- and macrovesicular lipidosis at the end of the 12-week HFD. The size bars represent 100µm (**a**) and 20µm (**b**).

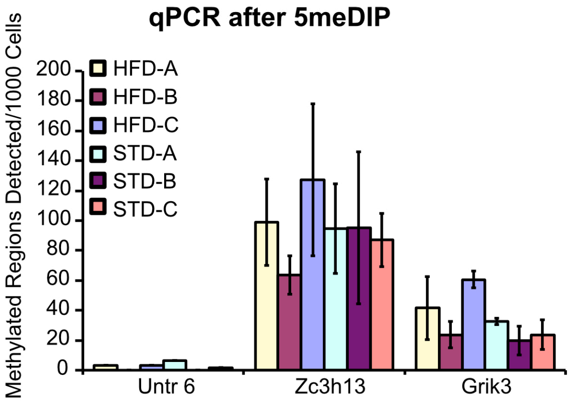

Figure S2. Successful pulldown of 5-methylcytosine DNA. Two known methylated sites at the *Zc3h13* and *Grik3* genes and one unmethylated negative control site termed “*Untr6*“ show the expected enrichments at the 2 positive control sites and low signals at the negative control site.

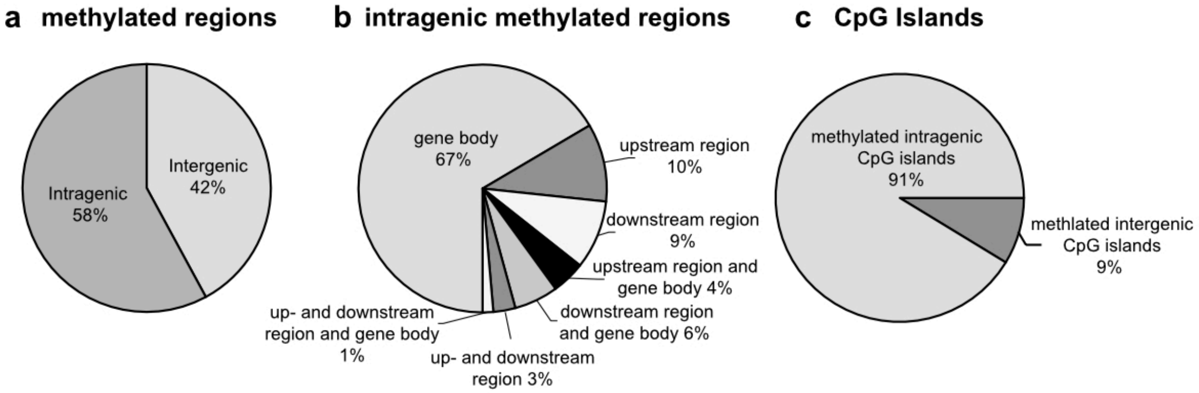

Figure S3. Distribution of methylated regions of the hepatic genome determined by 5meDIP-seq. (**a**) Intragenic and intergenic methylated regions. (**b**) Intragenic methylated regions within gene boundaries 10 kb up- and downstream of the first and last exon. (**c)** Intragenic and intergenic methylated CpG islands.

Table S1. Gene expression list

EdgeR determined differential gene expression in HFD livers versus STD livers (n = 3 pools of 2 livers / diet) based on quantification of transcript features by HTSeq against the GENCODE reference gtf file vM4 for the mouse mm10 reference genome. Alternative splicing was not considered a factor in the analysis. FC (H/S) is the fold change in HFD versus STD livers. P-value refers to the non-cutoff adjusted *p*-value. FDR is the False Discovery Rate-adjusted *p*-value. CPM is the average expression across all samples in counts per million.

Table S1 is available as .xlsx file (Supplementary Dataset 1).

Table S2. Differentially expressed genes and their flanking/overlapping lncRNA genes

EdgeR determined differential expression of 425 coding and non-coding genes with average expression levels > 1 CPM (columns A-G). DAVID and BiNGO/Cytoscape3.2 assigned biological processes to 390 and 350 DEG, respectively (columns H-I). 100 DEG were flanked or overlapped by at least one of 144 annotated lncRNA genes (columns J-M). Of these, 132 (92%) were not expressed or had extremely low average expression levels < 1 CPM, and 12 (8%) had average expression levels > 1 CPM (column M). FC (H/S), fold-change HFD versus STD. FDR, False Discovery Rate-adjusted *p*-value. CPM, counts per million. NA, not available.

Table S2 is available as .xlsx file (Supplementary Dataset 2).

Table S3. Validation of edgeR-calculated gene expression changes by nCounter® technology

Fold-changes of 95 protein-coding genes and 11 lncRNA genes determined by edgeR in liver pools (n = 3 pools of 2 livers / diet) (columns A-F) were validated by nCounter® technology in individual livers (n = 6 per diet) (columns G-I). nCounter probes targeted all or individual transcript isoforms (column I). Fold-changes refer to the levels in HFD livers relative to STD livers (FC (H/S), column G). Significant expression differences were determined by a two-sided *t* test (column H). FDR, False Discovery Rate-adjusted *p*-value. CPM, counts per million.

Table S3 is available as .xlsx file (Supplementary Dataset 3).

Table S4. Differentially expressed genes in overrepresented biological processes

DAVID and BiNGO/Cytoscape3.2 mapped DEG (columns A-B) to biological processes (columns C-D) and then determined statistically significant overrepresentation of biological processes. Several relevant DEG were not mapped by DAVID and BiNGO/Cytoscape3.2 and thus not considered for analysis. Determination of differential gene expression by edgeR (columns E-H) was validated by nCounter® technology. Expression differences were assessed by two-sided *t* test (columns I-J). FC (H/S), fold-changes in HFD livers relative to STD livers. edgeR *p*-value, edgeR-calculated non-adjusted *p*-value. edgeR FDR, edgeR-calculated False Discovery Rate-adjusted *p*-value. CPM, counts per million. n.d. , not determined. GO-ID, gene ontology identity number.

Table S4 is available as .xlsx file (Supplementary Dataset 4).

Table S5. Expression ranking of lncRNA genes

343 lncRNA genes expressed across all liver samples at levels > 1 CPM were ranked by expression.

Table S5 is available as .xlsx file (Supplementary Dataset 5).

Table S6. Co-expression of lncRNA genes with their nearest protein-coding genes

Co-expression of lncRNA genes (columns A-C) with their nearest protein-coding genes (column E-F, P) was ranked by the Pearson's correlation coefficient *r* (column H). An *r* value > 0.73 or < -0.73 was considered significant ($p < 0.05$, n = 6, one-tailed test). Expression of lncRNA and protein-coding genes was positively or negatively correlated (column I). LncRNA genes were positioned up- or downstream of protein-coding genes, overlapped their promoters (spanning sequence 10kb upstream of first exon), and /or gene bodies (columns K-L). The distance between lncRNA and protein-coding genes (column J) refers to the distance between their nearest borders irrespective of strand orientation (columns D, G) and was defined as 0 for overlapping lncRNA and protein-coding genes. Only lncRNA and protein-coding genes with average expression levels > 1 CPM were analysed (columns M-N). In most cases, expression levels of protein coding genes exceeded those of their flanking/overlapping lncRNA genes (column O). Gene ontologies of co-expressed protein-coding genes (column Q) suggest a possible involvement of lncRNAs in the regulation of various biological processes.

Table S6 is available as .xlsx file (Supplementary Dataset 6).

Table S7. Diet-responsive co-expression of lncRNA and protein-coding genes

4 (columns A-F) out of 14 differentially expressed lncRNA genes (Table 4) were co-expressed with 3 differentially expressed protein-coding genes *in cis* (*Gm26870/Gm10717*, *Gm11399/Sfi1*, *A530040E14rik/C130026I21Rik, Gm16025/C130026I21Rik*; columns G–K) as determined by a Pearson's correlation coefficient $r > 0.73$ or < -0.73 ($p < 0.05$, n = 6, one-tailed test; column L). Co-expression was concordant in all cases (column M). The distance between lncRNA and coding genes (column N) refers to the distance between their closest borders irrespective of strand orientation (columns D, I) and was defined as 0 for overlapping lncRNA and coding genes. LncRNA genes were positioned up- or downstream of the coding gene (column O), overlapped the coding gene body (column O), and/or the coding gene promoter (10kb sequence upstream of first exon; column P). Only lncRNA and coding genes with average expression levels > 1 CPM (columns Q-R) were analysed. Gene ontologies of co-expressed protein-coding genes (column U) suggest a possible involvement of lncRNAs in diet-induced cell division, transcription and chromatin remodelling. NA, not available.

Table S7 is available as .xlsx file (Supplementary Dataset 7).

Table S8. Methylated region statistics

Sample	Number of methylated regions
HFD A	96693
HFD B	87925
HFD C	59535
STD A	82862
STD B	78770
STD C	94627

Peaks in individual 5meDIP-seq samples were identified by SICER relative to input control DNA pooled from all samples as a reference and using the following parameters: significance threshold = FDR 1E-10, window size = 200 bp, gap size = 0 bp, fragment size = 150 bp. Overlapping peaks between samples were grouped into methylated regions whose margins were defined by the start coordinate of the most upstream peak and the end coordinate of the most downstream peak. When only one sample showed a peak, that peak defined the methylated region. The number of methylated regions in each sample varied between 59000 and 97000 from a total of 154664 methylated regions.

Table S9. Methylated regions

5meDIP-seq assessed cytosine methylation in liver DNA pools (n = 3 pools of 2 livers / diet) and revealed a total of 154664 methylated regions, which were assigned unique numerical identifiers (column A). The genomic locations of methylated regions (mm9 mouse reference genome) are indicated by chromosome number (column B), nucleotide start and end position on the +DNA strand (columns C-D) and length in base pairs (column E). Methylated regions are composed of a number of overlapping peaks (Peak Count, column F). The presence or absence of a methylated region in individual samples is denoted by "1" or "0", respectively (columns AI-AN). The average fragment counts across

a methylated region (Count Avg Value, columns G-L) in STD and HFD livers were used to determine fold-changes in methylation (column M) by the *t* test (column N). P-values corrected for multiple testing by the Bonferroni, Holm, Hochberg, Benjamini-Hochberg (BH), and Benjamini-Yekutieli (BY) methods (columns O-S) were not significant. The highest fragment counts (Count Peak Value, columns T-Y) of regions that tended to be differentially methylated ($p < 0.05$ by *t* test) were low (< 20). DESeq2 (columns AA-AB) confirmed the absence of differentially methylated regions since FDR-adjusted *p*-values (column AB) were not significant. Methylated regions overlapped a variable number of CpG islands (CG Island Counts, column AC), promoters (-7500 bp to +2500 bp relative to first exon) (Promotor Count, column AD), and/or genes (columns AE-AF). Please note that overlap determination with a gene included the 10 kb flanking sequence upstream and downstream of the gene's first and last exon, respectively. The distance between the midpoint of a methylated region and the first exon of a gene (Dist to Start, column AG) was negative if the methylated region was located upstream and positive if located downstream of the first exon. Methylated regions overlapped the upstream region, body or downstream region of a gene (column AH).

Table S9 is available as .xlsx file (Supplementary Dataset 9).

Table S10. CpG methylation

BS-seq assessed CpG site methylation in liver DNA pools (n = 3 pools of 2 livers / diet). We studied 10 methylated regions, which were embedded in or associated with protein-coding genes within margins of 10 kb (columns A-B) and tended to be differentially methylated by 5meDIP-seq (*p*-value < 0.05 by *t* test). These methylated regions have a unique numerical identifier (column C; see Table S9) and contain 4 – 16 CpG sites (columns D-G). The genomic coordinates of each CpG site are indicated by chromosome number (Chr, column E) and nucleotide position on the + DNA strand (column F)

within the mm9 mouse reference genome. Most CpG sites were strongly methylated in all liver DNA pools as shown by the high CpG methylation percentages (columns H-M). No or low CpG methylation is shown in blue, higher CpG methylation is shown in increased shades of red, while intermediate values are shown in white. While 9 methylated regions showed equal CpG methylation percentages in both diets (columns O-P), one methylated region within the *Wwc1* gene tended to have decreased CpG methylation percentages in HFD livers, as suggested by the *t* test (p-value < 0.05, column Q) and Mann-Whitney test (p-value = 0.1, column R). The alignment coverage for all CpG sites (columns T–Y) varied between samples and at different locations but was far over 1000 at most CpG sites.

Table S10 is available as .xlsx file (Supplementary Dataset 10).

Table S11. All C methylation

Assessment of the methylation percentages of both CpG and non-CpG sites (sequence context CHH and CHG, where H = A, T, or C; column C) for the 10 methylated regions (columns A-B) described in Table S10. The methylation percentage was below 1% for most non-CpG sites (columns G-L). Mean methylation percentages of most non-CpG sites (columns N-O) were the same in both diets, as determined by *t* test (column P) and Mann-Whitney test (column Q). Methylated regions are denoted by unique numerical identifiers (column B; see Table S9 and S10). The genomic coordinates of CpG and non-CpG sites are indicated by chromosome number (column D) and nucleotide position on the + DNA strand (column E) within the mm9 mouse reference genome.

Table S11 is available as .xlsx file (Supplementary Dataset 11).

Table S12. Putative Srebf1 and Srebf2 transcription factor binding sites

Differentially expressed genes mediating steroid and cholesterol synthesis, as well as fatty acid and triglyceride synthesis (columns A-D) had several putative binding sites for Srebf1 (columns E-F) and Srebf2 (columns G-H) in their promoters (-5000 bp to +1000 bp relative to the transcription start site (TSS)) as predicted by the EPD [1] search motif tool at a cutoff *p*-value of 0.001. Binding site sequences upstream of the TSSs are denoted in small letters while those located downstream of the TSSs are denoted in capital letters. Underlined sequences are shared predicted binding sites between Srebf1 and Srebf2. Please note that *Hmgcr* and *Pmvk* have alternative TSSs and promoters. A search in GTRD [2], a database of previous mouse ChIP-seq experiments, revealed a number of Srebf1 peaks called by 4 different peak callers (MACS, SISSRs, GEM and PICS) (columns I-L) in all examined genes. These Srebf1 peaks overlapped most gene promoters, as well as the 5'-untranslated regions (utr) and gene bodies (columns M-T), thus providing experimental evidence for the likely existence of Srebf1 binding sites in these genes. Bolded peak coordinates (columns M, O, Q, S) refer to peaks identified in liver samples. All coordinates relate to the mm10 mouse reference genome.

Table S12 is available as .xlsx file (Supplementary Dataset 12).

References to Supplementary Information

1 Dreos, R., Ambrosini, G., Groux, R., Cavin Perier, R. & Bucher, P. The eukaryotic promoter database in its 30th year: focus on non-vertebrate organisms. *Nucleic acids research* **45**, D51-D55 (2017).

2 Yevshin, I., Sharipov, R., Valeev, T., Kel, A. & Kolpakov, F. GTRD: a database of transcription factor binding sites identified by ChIP-seq experiments. *Nucleic acids research* **45**, D61-D67 (2017).
